# Prediction of functional microRNA targets by integrative modeling of microRNA binding and target expression data

**DOI:** 10.1186/s13059-019-1629-z

**Published:** 2019-01-22

**Authors:** Weijun Liu, Xiaowei Wang

**Affiliations:** 1 Department of Radiation Oncology, Washington University School of Medicine, St. Louis, MO USA; 2 Nawgen LLC, St. Louis, MO USA

**Keywords:** MicroRNA, Target prediction, RNA-seq, CLIP-seq

## Abstract

We perform a large-scale RNA sequencing study to experimentally identify genes that are downregulated by 25 miRNAs. This RNA-seq dataset is combined with public miRNA target binding data to systematically identify miRNA targeting features that are characteristic of both miRNA binding and target downregulation. By integrating these common features in a machine learning framework, we develop and validate an improved computational model for genome-wide miRNA target prediction. All prediction data can be accessed at miRDB (http://mirdb.org).

**Electronic supplementary material:**

The online version of this article (10.1186/s13059-019-1629-z) contains supplementary material, which is available to authorized users.

## Background

MicroRNAs (miRNAs) are small noncoding RNAs that are extensively involved in many diverse biological processes, and dysregulation of miRNA expression may lead to a variety of diseases [1]. To date, over 2000 human miRNAs have been reported in miRBase [2]. Both computational and experimental analyses indicate that most human protein-coding genes are regulated by one or more miRNAs [3–5]. For functional miRNA analysis, one critical first step is to identify genes targeted by the miRNA. To this end, most studies rely on computational tools to initially identify promising target candidates, which are subject to experimental validation at a later stage. Given the critical role of target prediction in miRNA functional characterization, many computational tools have been developed in the past 10 years, with gradually improved performance on target identification. In particular, in recent years, new models have been developed based on breakthroughs in experimental methods as well as novel insights into the mechanisms of miRNA target regulation [6]. Many common features have been discovered for miRNA target regulation, such as perfect pairing of the miRNA 5′-end (seed region) to the target site, as well as relatively low GC content of the target site, which results in increased site accessibility for miRNA binding [7–13].

Despite steady progress in the field of miRNA target prediction, available prediction algorithms still have suboptimal performance, leading to frequent false predictions that are experimentally costly at the validation stage. Thus, further improvement in computational target prediction is of high practical importance. However, efforts in model improvement are greatly hindered by the lack of high-quality training data from experimental studies. For computational target analysis, high-quality training data are essential not only to identify relevant target features but also to properly weight and combine these features for building the final prediction models. In fact, all commonly used target prediction algorithms were trained with various high-throughput profiling data, including microarray profiling data [14, 15], or more recently with crosslinking and immunoprecipitation (CLIP) sequencing data [16–18]. Of note, CLIP is able to identify transcript targets associated with functional miRNA-RNA-induced silencing complex (RISC) complex [19–21]. In a typical CLIP experiment, short transcript sequences that are bound to the Ago protein are identified by crosslinking the target RNA to the RISC protein complex, followed by immunoprecipitation and high-throughput RNA-seq analysis [20]. Recent improvements in CLIP studies further allow unambiguous identification of paired miRNA-target transcripts that reside in the same RISC complex by direct ligation of the miRNA and its cognate target transcript [22, 23]. Although CLIP data have been widely used to train miRNA target prediction models, one major concern is that miRNA target binding, as revealed by CLIP, does not necessarily result in functional target suppression [15, 24]. Thus, a large number of predicted miRNA targets based on CLIP training data may not be functionally relevant in gene expression regulation.

Besides CLIP-seq, another popular strategy for target analysis is to identify downregulated transcripts resulting from miRNA overexpression [3, 25, 26]. Targets identified in this way are more likely to be functionally relevant as implied by significant expression downregulation. However, there are also concerns about the miRNA overexpression strategy, as it is often challenging to distinguish direct miRNA targets from indirect targets (i.e., genes that are indirectly downregulated due to suppression of direct targets). Another concern is that some targets identified under miRNA overexpression in cell culture may not be physiologically relevant. Furthermore, miRNA overexpression analysis is also greatly limited by the lack of high-quality transcriptome-wide profiling data. Specifically, most existing datasets are of small scale, focusing only on a few miRNAs in any single study, and thus are not ideal for training a general target prediction model. Although it is possible to combine data from multiple small-scale studies, significant heterogeneity among different experiments poses a major concern for accurate target modeling. Despite the aforementioned challenges, microarray data from miRNA overexpression studies have been proven valuable for target analysis and have been used to train several widely used target prediction models [14, 15].

In this study, we analyzed both CLIP binding data and miRNA overexpression data to identify common features that are characteristic of both miRNA binding and target downregulation. As the first step, we performed a large-scale miRNA overexpression study that is specifically designed to identify transcripts downregulated by 25 individual miRNAs. To our knowledge, this is the largest RNA-seq study of its kind for miRNA target identification. This comprehensive dataset allowed us to quantitatively re-characterize the previously reported features in the context of target downregulation at the transcriptome level. miRNA targeting features identified from overexpression data were also compared to those identified from public CLIP binding data, and both datasets were integrated into the same machine learning framework for model development. In this way, our final target prediction model, MirTarget v4.0, possesses common features that are important for both miRNA binding and functional target downregulation. Comparative analysis indicates that MirTarget has improved performance over other state-of-the-art target prediction tools.

## Results

### RNA-seq to identify transcripts downregulated by miRNA overexpression

It is well established that the binding of a miRNA to its target transcript does not necessarily result in gene expression downregulation. In fact, most observed miRNA binding events, as revealed by CLIP analysis, have little functional consequences [15, 24]. Thus, focusing on miRNA binding alone has limited value for the prediction of functional miRNA targets, i.e., downregulated targets. To alleviate this concern, we directly determined the target downregulation by miRNA with RNA-seq. The overall study design is summarized in Additional file 1: Figure S1. As the first step, 25 miRNAs, along with a negative control RNA, were individually overexpressed in HeLa cells by transfection. These 25 miRNAs are listed in Table 1. The impact of miRNA overexpression was profiled at the transcriptome level by RNA-seq experiments. To control for experimental variations, each miRNA was transfected into cells in duplicate on different days, and RNA-seq library construction and sequencing runs were also performed in duplicate on different days. In total, 1.5 billion reads were generated for expression profiling of 52 RNA samples. The profiling data are presented in Additional file 2: Table S1. All sequencing data were combined to identify the genes downregulated by miRNA overexpression. In our analysis, transcripts that contain at least one miRNA seed binding site and were downregulated by at least 40% in both of the duplicated experiments are designated as miRNA targets. In contrast, transcripts that contain at least 1 seed site but had no expression change are designated as non-target controls. In this way, 2240 and 4127 miRNA targets and non-target controls were identified by RNA-seq, respectively. Specifically, there were 90 targets identified for each miRNA on average, and the target numbers vary considerably among individual miRNAs (ranging from 11 to 206, Table 1).

**Table 1:** Twenty-five miRNAs analyzed in the RNA-seq experiments

miRNA name	miRNA sequence	Identified targets
hsa-let-7c-5p	UGAGGUAGUAGGUUGUAUGGUU	31
hsa-miR-107	AGCAGCAUUGUACAGGGCUAUCA	35
hsa-miR-10a-5p	UACCCUGUAGAUCCGAAUUUGUG	32
hsa-miR-124-3p	UAAGGCACGCGGUGAAUGCC	151
hsa-miR-126-3p	UCGUACCGUGAGUAAUAAUGCG	11
hsa-miR-126-5p	CAUUAUUACUUUUGGUACGCG	48
hsa-miR-133b	UUUGGUCCCCUUCAACCAGCUA	108
hsa-miR-142-3p	UGUAGUGUUUCCUACUUUAUGGA	108
hsa-miR-145-5p	GUCCAGUUUUCCCAGGAAUCCCU	82
hsa-miR-146a-5p	UGAGAACUGAAUUCCAUGGGUU	42
hsa-miR-155-5p	UUAAUGCUAAUCGUGAUAGGGGU	154
hsa-miR-15a-5p	UAGCAGCACAUAAUGGUUUGUG	108
hsa-miR-16-5p	UAGCAGCACGUAAAUAUUGGCG	122
hsa-miR-17-5p	CAAAGUGCUUACAGUGCAGGUAG	74
hsa-miR-193b-3p	AACUGGCCCUCAAAGUCCCGCU	102
hsa-miR-200a-3p	UAACACUGUCUGGUAACGAUGU	35
hsa-miR-200b-3p	UAAUACUGCCUGGUAAUGAUGA	126
hsa-miR-200c-3p	UAAUACUGCCGGGUAAUGAUGGA	93
hsa-miR-206	UGGAAUGUAAGGAAGUGUGUGG	206
hsa-miR-210-3p	CUGUGCGUGUGACAGCGGCUGA	43
hsa-miR-21-5p	UAGCUUAUCAGACUGAUGUUGA	11
hsa-miR-31-5p	AGGCAAGAUGCUGGCAUAGCU	85
hsa-miR-34a-5p	UGGCAGUGUCUUAGCUGGUUGU	155
hsa-miR-9-3p	AUAAAGCUAGAUAACCGAAAGU	182
hsa-miR-9-5p	UCUUUGGUUAUCUAGCUGUAUGA	106

### The impact of miRNA seed types on target downregulation

Previous studies have identified several major types of canonical miRNA target sites, including those matching to the 6-mer, 7-mer, or 8-mer miRNA seed sequences (Table 2). Sequence conservation analysis suggested that target sites pairing to longer miRNA seeds are more conserved across species and thus are more likely to be bona fide miRNA targets [27]. This hypothesis on the seed type strength has also been confirmed by analyzing heterogeneous microarray datasets in the context of target downregulation [15, 28]. However, further analysis is needed to accurately quantify the contribution of each seed type. Our newly generated large-scale RNA-seq dataset, encompassing 25 miRNAs assessed under uniform experimental conditions, provided a unique opportunity to quantitatively evaluate the strength of different miRNA seeds on target downregulation. Specifically, we evaluated the enrichment of each seed type in downregulated target sites as compared to non-target sites.

**Table 2:** Enrichment of miRNA seed match in the target sites

Seed type	Matching positions in miRNA	Downregulated targets	Non-targets	Enrichment ratio
Seed6	pos 2–7	0.86	0.36	2.40
Seed7a	pos 1–7	0.46	0.13	3.45
Seed7b	pos 2–8	0.62	0.15	4.18
Seed7A1	pos 2–7 + A at target pos 1	0.52	0.13	4.10
Seed8	pos 1–8	0.26	0.05	5.48
Seed8A1	pos 2–8 + A at target pos1	0.30	0.04	6.83
Seed7b_not_U	Exclude miRNAs with 5′-U	0.60	0.15	3.97
Seed8_not_U	Exclude miRNAs with 5′-U	0.19	0.06	3.32
Seed8A1_not_U	Exclude miRNAs with 5′-U	0.34	0.05	6.95

**Fig. 1:**
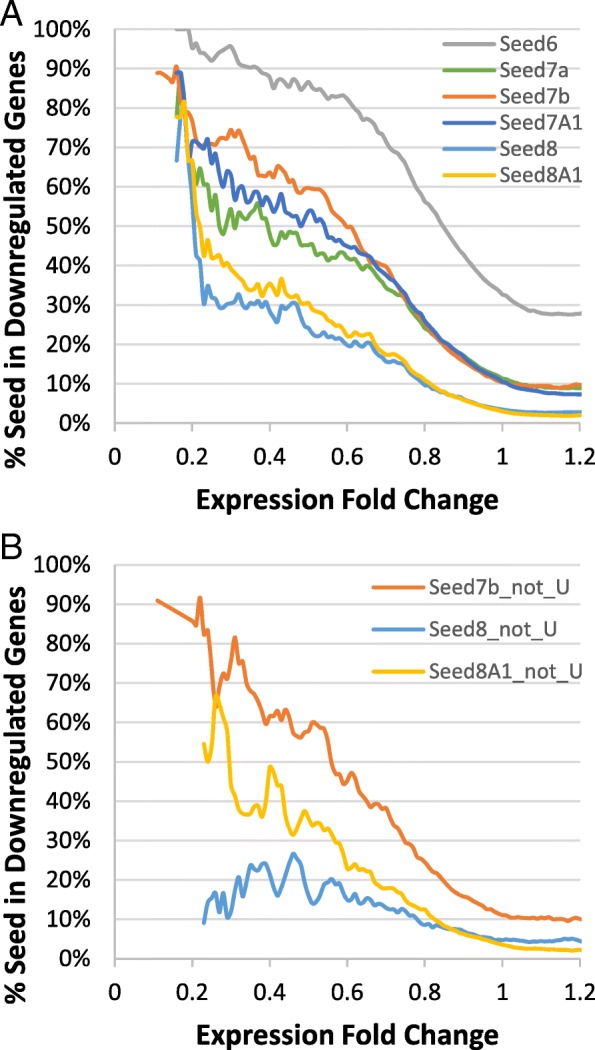
The impact of miRNA seed types on target downregulation. Six seed types were evaluated (see Table 2 for seed definitions). **a** Percentage of downregulated genes containing individual seed types in relation to gene expression changes. All 25 miRNAs were included in the analysis. **b** Analysis of a subset of 8 miRNAs that do not contain 5′-U

As shown in Table 2 and Fig. 1a, seed6 is the most prevalent type, identified in 86% of all downregulated targets. However, due to its short length, seed6 is also present non-specifically in 36% of non-target sites, resulting in the lowest seed enrichment ratio (2.40 in Table 2). On the other end, seed8A1 is the most selective type, with an enrichment ratio of 6.83 and is present in 30% of downregulated targets. Among all 7-mer seeds, seed7b and seed7A1 have similar enrichment ratios, both of which are higher than the ratio for seed7a.

Another type of 8-mer seed, seed8, has the second highest enrichment ratio of 5.48, which is higher than the ratios for all 7-mer seeds. To further distinguish the potential contribution of the terminal base match from terminal A base in the target site, we exclusively focused on 8 miRNAs that do not have a 5′-end U (Fig. 1b). When compared with all 25 miRNAs, we observed similar enrichment ratios for seed7b and seed8A1, respectively, from this subset of miRNAs (Table 2). These results suggest that terminal A-U perfect match has little impact on target recognition, as the presence of terminal A in target sites, regardless of its pairing status to the miRNA, is associated with target downregulation. Interestingly, we also observed a dramatically decreased enrichment ratio for seed8 from this miRNA subset. In fact, the seed8 ratio (3.32) is even lower than that for seed7b (Table 2). Thus, a perfect terminal match other than A-U is detrimental (rather than contributing) to target recognition. Based on the seed analysis, we decided to focus on 3 strongest seed types, including seed8A1, seed7b, and seed7A1, for target prediction modeling. Combined together, these 3 seed types were identified in the 3′-UTR of 76% of downregulated transcripts.

### Combining target downregulation and CLIP binding data to identify common targeting features

One common concern with miRNA overexpression studies is that it is challenging to locate the exact miRNA binding site within the target transcript. To alleviate this concern, we identified candidate target sites based on the presence of canonical 7-mer or 8-mer seed sites. In contrast to miRNA overexpression analysis, CLIP-ligation studies are able to unambiguously identify miRNA binding sites in the target transcript by crosslinking the miRNA and its cognate target site in the same RISC complex. However, the functional consequence of miRNA target binding, as identified by CLIP, cannot be easily determined. Thus, both CLIP binding and miRNA overexpression methods have pros and cons, and each method alone depicts only one important aspect of miRNA target regulation, i.e., target binding and functional suppression, respectively.

In our analysis, we are interested in identifying common features that are characteristic of functional target regulation, including both miRNA binding and subsequent target downregulation. In a recent target prediction analysis [18], we have compiled a miRNA target binding dataset derived from multiple public CLIP ligation studies [22, 23]. The CLIP ligation method is considered advantageous over traditional CLIP methods, as both the miRNA and its cognate binding site in the target transcript can be unambiguously identified by crosslinking to the same RISC complex. In the present study, the CLIP binding dataset was further combined with new miRNA overexpression data to identify targeting features that are common to both miRNA binding and target suppression. In this way, 4774 target sites and 8081 non-target sites, identified from both CLIP and miRNA overexpression studies, were combined and evaluated in subsequent feature analysis.

Target and non-target sites in the combined dataset were compared to identify the features that are commonly associated with miRNA target regulation. These features are listed in Additional file 3: Table S2. It is well-established that miRNA target sites are evolutionarily conserved [7, 28]. In our study, we evaluated target conservation using two complementary approaches. First, we calculated the difference in conservation scores between seed binding positions and flanking positions, as determined by phyloP scores from 100-way multi-genome alignment [29]. Second, we also determined whether the entire seed site (7-mer or 8-mer) is found across multiple species by word search. Both conservation analyses indicated that target sites were very significantly conserved as compared to non-target sites. In fact, seed conservation was among the most significantly enriched features, whether miRNA overexpression and CLIP binding data were analyzed separately, or in combination. Specifically, conserved seed8A1 was the most enriched in target sites (*p* = 2.8E−245 by cross-species seed match and *p* = 7.3E−218 by phyloP score, respectively). On the other end, non-conserved seed7A1 was the most depleted seed type (9.5E−134 by seed match and *p* = 1.3E−138 by phyloP score, respectively). Besides seed conservation, there were many other features commonly found in both datasets. For example, miRNA target sites were preferentially associated with shorter 3′-UTR sequences (*p* = 4.7E−126), and they were more likely to be found toward the end of the 3′-UTR sequence (*p* = 5.4E−66) and away from the center of long transcripts (*p* = 2.5E−87).

Despite many similarities, there are also distinct differences between miRNA overexpression and CLIP binding data. One prominent example is related to the GC content of the target site. Compared to non-target sites, target site GC content was much lower in CLIP binding data (*p* = 1.9E−146), but only modestly lower in miRNA overexpression data (*p* = 2.1E−10). The depletion of C nucleotide was moderate in both datasets. Thus, the drastic difference in GC content between the two datasets was mainly the result of a much stronger bias against G nucleotide in the CLIP data (*p* = 7.7E−137), in contrast to the overexpression data (*p* = 1.2E−19). One possible explanation could be related to RNase T1 used in CLIP studies, which preferentially cuts at G nucleotide, resulting in the depletion of internal G in sequencing reads. However, it could also be true that enrichment of G hinders target site binding by the miRISC complex, as G was also depleted in miRNA overexpression data, although only moderately. Another interesting feature is the seed binding stability, as determined by the free energy of the seed/target duplex. Seed binding stability was favored in miRNA overexpression data (*p* = 2.5E−12), but disfavored in CLIP binding data (*p* = 5.4E−26). Overall, this feature was no longer significant when the two datasets were combined (*p* = 0.26).

### Developing a target prediction model with common targeting features

All miRNA targeting features, as listed in Additional file 3: Table S2, were modeled in a support vector machine (SVM) framework for algorithm development. Furthermore, we also performed recursive feature elimination (RFE) analysis to rank the relative importance of each feature for its independent contribution to model performance. In this RFE evaluation, all the features were analyzed collectively using SVM. Specifically, as the first step, the least important feature was identified and subsequently removed from the model. Next, the remaining features were evaluated to identify the second least important feature for elimination. This evaluation process was repeated with one feature eliminated from each iteration until only one feature remained. The RFE approach helps to understand the independent contribution of individual features that are included in the model. Table 3 summarizes 20 top-ranking targeting features by RFE analysis. The complete RFE ranks of all the features are listed in Additional file 3: Table S2. Consistent with the feature analysis presented in the previous section, multiple seed conservation features ranked among the highest by RFE analysis, with conserved seed8A1 as the most impactful feature. In our final SVM model, all 96 features, including both statistically significant and non-significant ones, were integrated for building the prediction model, which we named MirTarget v4.0. Fivefold cross-validation was performed to determine the optimal parameters for the SVM kernel function using the grid.py tool in the libsvm package. A scoring scheme was then developed to represent the confidence of prediction. For each candidate target site, MirTarget computes a probability score (in the range of 0–1) derived from the SVM modeling tool, libsvm, as previously described [30]. This target site score reflects the statistical assessment of the prediction accuracy. Based on individual target site scores, MirTarget predicts whether a gene is a miRNA target by combining all site scores within the 3′-UTR using the following formula:

$$ S=100\times \left(1-\underset{i=1}{\overset{n}{\Pi}}{P}_i\right) $$

where *n* represents the number of candidate target sites in the 3′-UTR, and *P*_*i*_ represents the probability score for each site as estimated by MirTarget. Most target genes contain only one site, and thus, the final target score is computed using the same equation with *n* = 1. MirTarget scores were used to rank the relative significance of the predicted targets. In this way, we employed MirTarget for genome-wide prediction of miRNA targets. All predicted targets are presented in miRDB (http://mirdb.org) [31].

**Table 3:** Summary of top-ranking miRNA targeting features identified by RFE analysis

Feature name	RFE rank	Targets	Non-targets	*P* value
Seed 8A1, conserved	1	0.184	0.018	2.8E−245
Seed7b, low phyloP score	2	0.273	0.445	3.2E−84
GC content of target site	3	1.554	1.901	4.9E−117
UTR length (log2)	4	10.960	11.430	1.5E−114
Seed7A1, non-conserved	5	0.142	0.341	9.5E−134
Seed7A1, low phyloP score	6	0.137	0.339	1.3E−138
AG count	7	0.517	0.774	2.6E−73
Seed8A1, low phyloP score	8	0.200	0.126	1.5E−29
Pentamer motif match	9	0.052	0.022	2.2E−19
Free energy of seed binding (log2)	10	− 2.583	− 2.596	2.6E−01
Distance to UTR end (log2)	11	8.403	9.125	4.7E−126
Seed8A1, moderate phyloP score	12	0.047	0.006	7.5E−53
CA count	13	0.758	0.743	2.7E−01
Seed7b, conserved	14	0.124	0.048	8.7E−55
Seed8A1, high phyloP score	15	0.146	0.009	7.3E−218
Seed7A1, high phyloP score	16	0.036	0.014	1.0E−15
Seed7b, high phyloP score	17	0.093	0.022	8.7E−74
CT count	18	0.893	0.829	3.5E−05
CG count	19	0.106	0.128	6.4E−04
TA count	20	0.871	0.655	2.1E−45

### Algorithm evaluation with independent experimental data

One common concern in algorithm development is that a model may work well on the training data, but not as well on independent unseen data. Thus, the best way to evaluate the performance of MirTarget would be to apply it to independent experimental data. In the present study, heterogeneous experimental data were analyzed for algorithm evaluation, including those generated from both CLIP binding and miRNA knockdown experiments. The performance of MirTarget was also compared to four other well-established algorithms, including TargetScan 7.0, DIANA-MicroT, miRanda (mirSVR), and PITA. These algorithms are among the most popular miRNA target prediction tools, and transcriptome-wide prediction data are readily downloadable from the respective websites.

#### Validation with CLIP-seq data

Chi et al. pioneered the HITS-CLIP method for experimental identification of miRNA target transcripts [20]. With this method, they performed crosslinking immunoprecipitation to pull down mRNA transcripts that were associated with the miRISC in mouse brain. High-throughput sequencing was then performed to identify these mRNA transcript tags, i.e., short RNA fragments protected by Ago from RNase digestion. Chi et al. demonstrated that in general, the transcript tags are centered on the seed binding sites [20]. This HITS-CLIP dataset was further analyzed in our study to identify potential miRNA target sites. Altogether, 886 potential target sites were identified based on the seed-matching sequences for the six most abundantly expressed miRNAs. As negative controls, a set of potential non-target sequences was also selected based on the following criteria: (1) they do not overlap with any sequence tags identified in the HITS-CLIP experiment and (2) they are from transcripts with detectable expression levels as revealed by microarrays. From these non-target sites, 889 with seed-matching sequences were selected as negative controls.

**Fig. 2:**
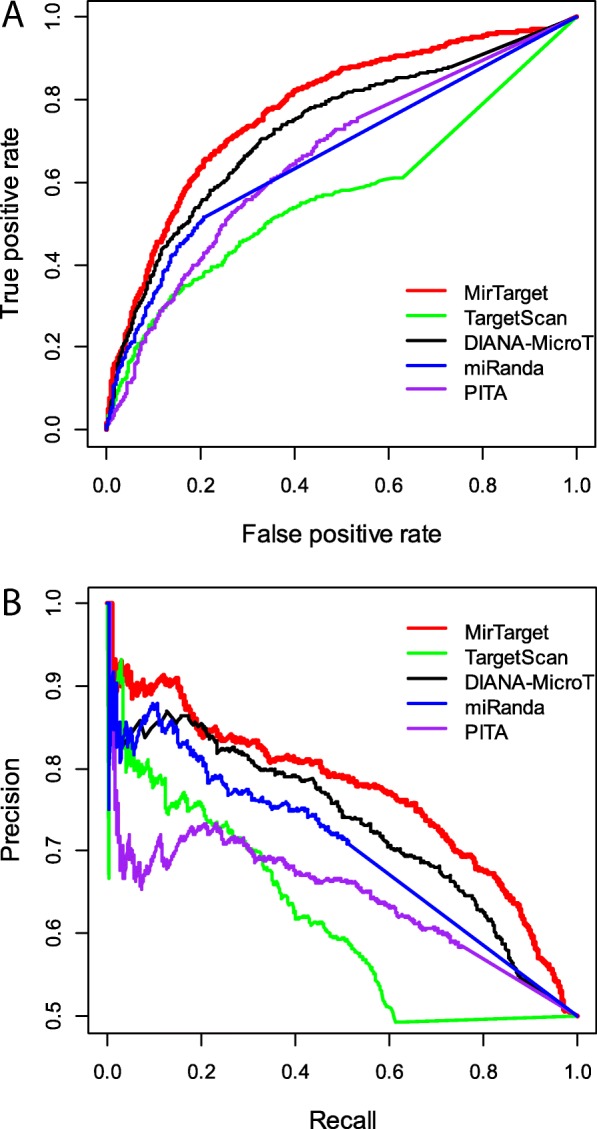
Comparison of miRNA target prediction algorithms using the HITS-CLIP dataset. MirTarget and four other target prediction algorithms were included in the analysis. **a** Receiver operating characteristic (ROC) curve analysis to evaluate the rate of false positive prediction in relation to the rate of true positive prediction. **b** Precision-recall (PR) curve analysis to evaluate prediction precision in relation to the recall rate

In our analysis, the performance of five computational algorithms, including MirTarget, TargetScan, DIANA-MicroT, miRanda, and PITA, was evaluated by comparing their ability to distinguish targets from non-targets as revealed by HITS-CLIP. ROC analysis was performed to evaluate the overall sensitivity and specificity of the prediction algorithms. As shown in Fig. 2a, MirTarget has the best performance, with an area under the ROC curve (AUC) of 0.78. DIANA-MicroT has the second best performance (AUC = 0.73). Interestingly, DIANA-MicroT was developed by training with CLIP binding data, whereas other public algorithms were trained with miRNA overexpression data. Thus, it is not surprising that DIANA-MicroT fits relatively well on CLIP testing data. Beside ROC analysis, we also constructed precision-recall (PR) curves to evaluate the accuracy of prediction. PR curves are commonly used in algorithm evaluation to determine prediction precision (proportion of true positives among all predicted positives) in relation to the recall rate (proportion of identified true positives among all true positives). As shown in Fig. 2b, MirTarget has the best performance among all five algorithms. In particular, the precision for MirTarget is over 90% when the recall rate is below 20%. This indicates that MirTarget is especially accurate for high-confidence predictions (i.e., high prediction scores).

#### Validation with miRNA knockdown data

**Fig. 3:**
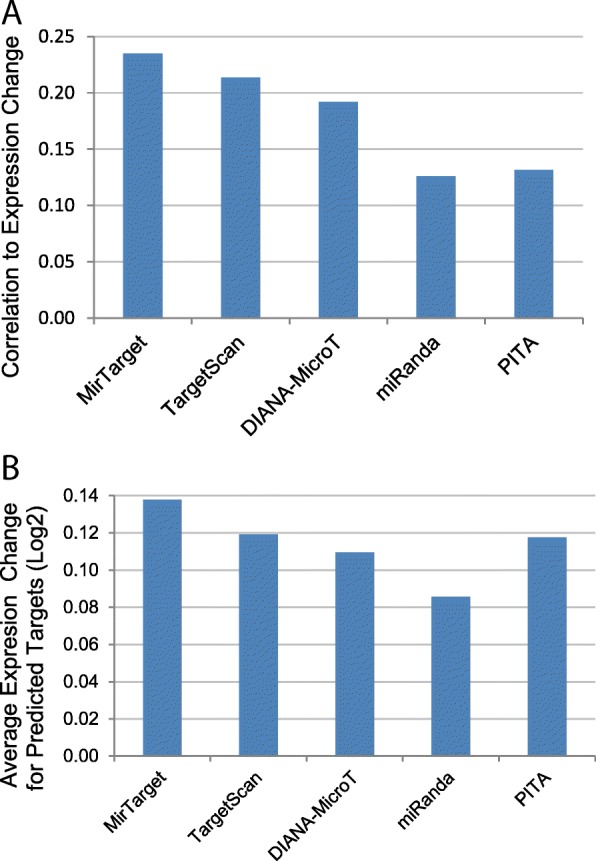
Comparison of target prediction algorithms using microarray data. Microarray profiling data were analyzed to identify target upregulation resulting from concurrent inhibition of 25 miRNAs. **a** Correlation of target upregulation and target prediction scores computed by 5 individual algorithms, as measured by Pearson correlation coefficient. **b** Average level of expression upregulation for predicted targets. For each algorithm, 100 top-scoring predicted targets per miRNA on average were included in the analysis

Target prediction algorithms were also evaluated in the context of target expression changes. In this comparative analysis, we evaluated the algorithms by employing a public miRNA knockdown study by Hafner et al. [21]. In that public study, the authors concurrently suppressed the functions of 25 miRNAs by antisense inhibitors and evaluated the impact on target RNA expression with microarrays. Genes targeted by these miRNAs were expected to be upregulated due to miRNA inhibition. In our analysis, we evaluated the correlation between target prediction scores and target expression upregulation. As shown in Fig. 3a, compared to other algorithms, the prediction scores computed by MirTarget have the highest correlation to gene expression upregulation. Furthermore, we also assessed gene expression changes for top-ranking predictions by individual algorithms, as researchers are particularly interested in high-confidence target candidates. To this end, we evaluated 100 top-ranking predicted targets per miRNA on average by each algorithm. Consistent with the correlation analysis, the targets predicted by MirTarget were upregulated the most on average as compared to those predicted by other algorithms (Fig. 3b).

## Discussion

Progress in miRNA target prediction is largely dependent on the availability of high-quality training datasets. In recent years, the advent of innovative CLIP-seq methods allows us to directly identify target transcripts that are bound to the miRISC complex. Although very useful, there are also concerns when CLIP data are applied to the training of target prediction algorithms. One major concern is that most targeting binding events observed in CLIP experiments have a little functional impact, as measured by target expression changes [15, 24]. It is likely that many transcripts identified by CLIP are only transiently recognized by miRISC but soon dissociated from it, without resulting in expression changes. It is also possible that binding by miRISC is functionally relevant in ways other than target downregulation, such as impacting the cytoplasmic distribution of miRNAs. For most miRNA studies, the researchers are interested in identifying target transcripts that are downregulated by the miRNA of interest. Thus, in the present study, we have combined CLIP binding data with miRNA overexpression data to systematically identify functional miRNA targets. Compared to CLIP studies, it is possible that overexpression of miRNAs may distort target regulation under normal physiological conditions. Thus, both CLIP and miRNA overexpression have major advantages and disadvantages for miRNA target analysis. Based on our analysis, CLIP binding and miRNA overexpression data share many common features, especially those related to seed conservation. However, we also observed significant differences in certain features, indicating that the two processes reflect different aspects of miRNA target regulation. We believe that, by modeling with both types of data, the prediction algorithm can be more generally applied to various experimental settings.

In the present study, we have experimentally generated a large RNA-seq dataset to study the functional impact of individual miRNAs. To our knowledge, our dataset, including 1.5 billion reads from 52 RNA samples, is the largest of its kind for miRNA target analysis. The newly generated RNA-seq dataset is not only crucial for this study but also enables further algorithmic improvement in future studies by us as well as other researchers in this field.

## Conclusions

We have developed a new miRNA target prediction algorithm, MirTarget, by combining CLIP binding and target downregulation data. Comparative analysis showed that MirTarget has improved performance over existing algorithms when applied to independent experimental data. All the target prediction data can be accessed at miRDB (http://mirdb.org) [31].

## Materials and methods

### RNA-seq experiments

RNA-seq was performed to evaluate the impact on the transcriptome by individual miRNAs. Specifically, each miRNA mimic (Nawgen) as well as a negative control RNA was individually transfected into HeLa cell with Lipofectamine 2000 (Life Technologies). Total RNA was then isolated 24 h post-transfection with mirVana kit (Life Technologies) for transcriptome analysis by RNA-seq. Details of the RNA-seq experimental protocol has been described previously [32]. In brief, ribosomal RNA was first removed using the RiboMinus kit (Life Technologies) and custom-designed oligonucleotide probes. Then, the RNA was used to construct RNA-seq libraries with the NEBNext mRNA Library Prep kit (New England Biolabs). The resulting cDNA libraries were PCR amplified with indexed primers and subject for sequencing with Illumina HiSeq 3000 at the Genome Technology Access Center of Washington University. In total, 1.5 billion reads were generated and each RNA sample received a coverage of 27 million raw sequence reads (50 n.t.) on average after demultiplexing the sample index. These raw reads were mapped to the human transcriptome with Bowtie [33] and then normalized by computing the gene expression count per million reads, followed by trimmed median normalization. A floor value of 5 was set for low readings (< 5). Normalized read counts from the miRNA overexpression samples were compared to those from negative control as well as other miRNA overexpression samples to identify gene expression changes at the transcriptome level. A gene was denoted as a miRNA target if, compared to the median of all samples, its expression level was reduced by at least 40%; a gene was denoted as a non-target if its gene expression level was at least 100%, but no more than 110% of the median.

### Public data retrieval

#### CLIP data

Details on CLIP-ligation data retrieval were described previously [18]. In brief, we collected and combined the data from the Helwak study [22] and the Grosswendt study [23]. Raw RNA-seq data from the Helwak study were downloaded from the NCBI GEO database (accession# GSE50452) [34]. Lists of curated miRNA/target pairs were downloaded from the journals’ website [22, 23]. The HITS-CLIP data [20] were downloaded from http://ago.rockefeller.edu. Raw sequence tags were aligned to the transcriptome with BLAT [35].

miRNA sequences were downloaded from miRBase [2]. RefSeq transcript sequences and related gene mapping index files were downloaded from NCBI [36]. The NCBI HomoloGene database [36] was used to map orthologous gene relationships across species. Basewise conservation was determined by computing phyloP conservation scores with PHAST [29] and downloaded from UCSC Genome Browser (https://genome.ucsc.edu/). miRNA target prediction data generated by public tools were retrieved from the respective websites (TargetScan 7.0 [15], http://targetscan.org; DIANA-MicroT [16], http://diana.imis.athena-innovation.gr; miRanda-mirSVR [14], http://microrna.org; PITA [13], https://genie.weizmann.ac.il/pubs/mir07/). The target transcript IDs from all the algorithms were mapped to NCBI Gene IDs for direct comparison.

#### Microarray data

We retrieved the microarray data reported by Hafner et al. [21]. In this microarray analysis, 25 miRNAs were inhibited by antisense oligonucleotide inhibitors, and the impact on gene expression was assessed with Affymetrix Human U133Plus2 chips. Raw microarray data were downloaded from the NCBI GEO database (accession# GSE21577), and then normalized using the Bioconductor RMA method (http://www.bioconductor.org). We focused our analysis only on genes with detectable expression. Changes in gene expression due to miRNA inhibition were determined by comparing to the negative controls.

### Computational data analysis

Statistical analysis was mainly performed with the R package (http://www.r-project.org/). Statistical significance for individual miRNA targeting features was calculated with Student’s *t* test or *χ*^2^ test. LIBSVM was used to train miRNA target prediction models based on the support vector machines (SVMs) (http://www.csie.ntu.edu.tw/~cjlin/libsvm/). For the SVM analysis, radial basis function (RBF) was used for kernel transformation. The RBF kernel parameters were optimized with grid search and cross-validation according to the recommended protocol by LIBSVM. We also performed recursive feature elimination (RFE) analysis with Weka (http://www.cs.waikato.ac.nz/ml/weka/) to evaluate the independent contribution of each feature in the SVM model.

## Additional files

Additional file 1: **Figure S1.** Overall study design for developing a new algorithm for miRNA target prediction. (PDF 6 kb)

Additional file 2: **Table S1.** RNA-seq profiles for miRNA overexpression. (XLSX 7339 kb)

Additional file 3: **Table S2.** Summary of miRNA targeting features. (XLSX 32 kb)
