# Supplementary figures and images for: Prediction of functional microRNA targets by integrative modeling of microRNA binding and target expression data
Source: Genome Biol. 2019 Jan 22;20:18. doi: 10.1186/s13059-019-1629-z (PMC6341724; doi:10.1186/s13059-019-1629-z)

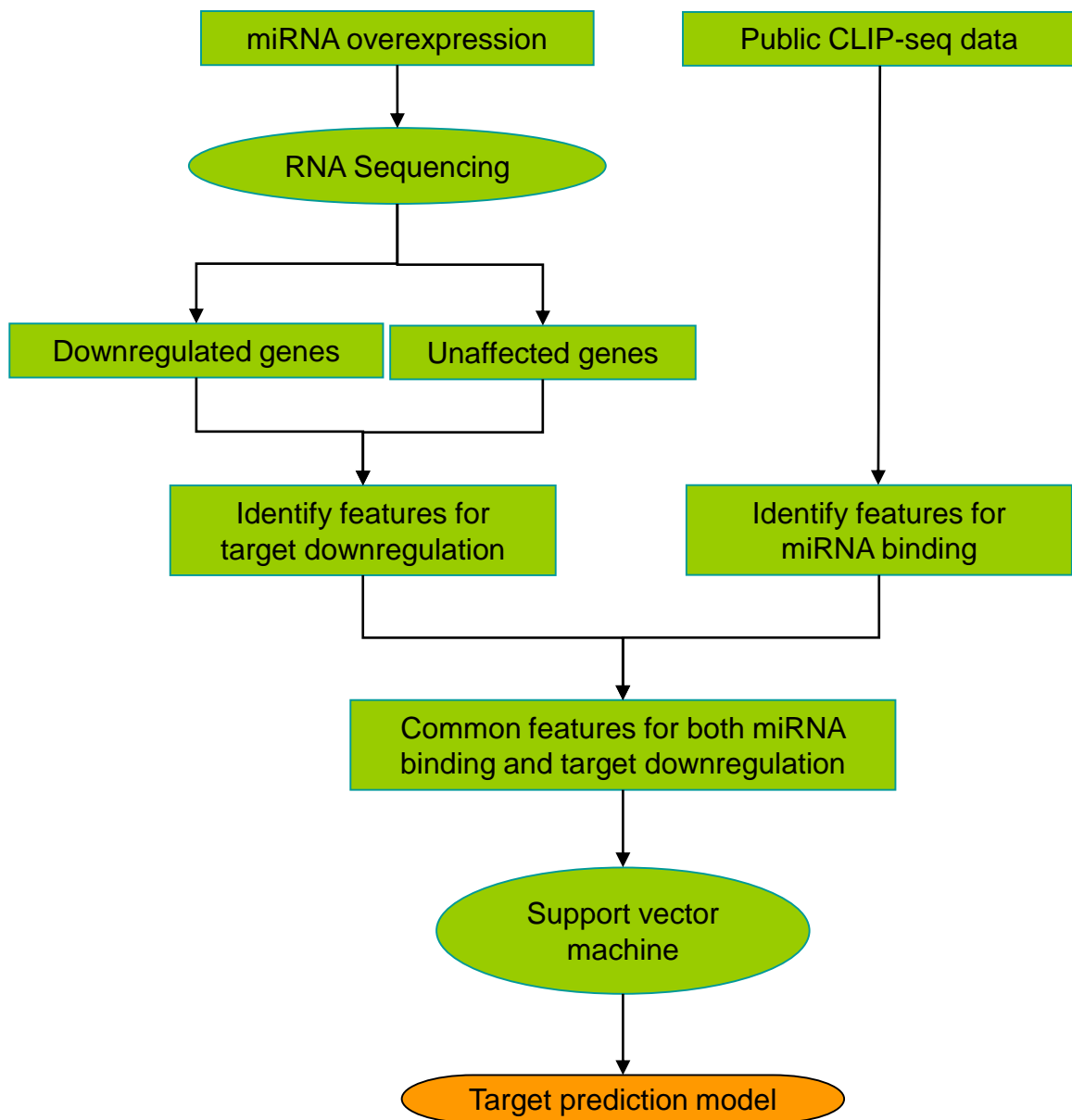

**Figure S1.** Overall study design for developing a new algorithm for miRNA target prediction.

Supplement: Supplementary file 1 — Figure S1. Overall study design for developing a new algorithm for miRNA target prediction. (PDF 6 kb) [file 13059_2019_1629_MOESM1_ESM.pdf]
